# Topical steroid withdrawal treated with ruxolitinib cream

**DOI:** 10.1016/j.jdcr.2024.03.011

**Published:** 2024-03-29

**Authors:** Moira Shea, Erin Grinich, Eric Simpson

**Affiliations:** Department of Dermatology, Oregon Health & Science University, Portland, Oregon

**Keywords:** allergic contact dermatitis, ruxolitinib, topical corticosteroids, topical steroid withdrawal

## Introduction

Topical steroid withdrawal (TSW) is an adverse effect caused by prolonged inappropriate use of topical corticosteroids (TCS), causing erythematous edematous well-demarcated plaques and severe burning sensations upon TCS discontinuation.^1^^,^^2^ Other clinical features include diffuse desquamation and a rosacea-like pattern.^1^^,^^3^ Although descriptions of this disease continue to emerge, less is known about appropriate treatment. Current recommendations in literature include 1 or more of the following: discontinuation of TCS with a possible slow taper, oral antihistamines, oral antibiotics, ice/cool compresses, or psychological support.^3^ Some online recommendations (itsan.org accessed December 10, 2023) suggest that healing is only possible through complete immediate cessation of TCS. We report the case of a 69-year-old woman with a multiyear history of burning, itching erythematous patches of the lips and cheeks who was diagnosed with TSW. The patient poorly tolerated tacrolimus 0.1% ointment and pimecrolimus 1% cream but her condition cleared with topical ruxolitinib 1.5% cream use.

## Case report

In 2022, a 69-year-old woman sought a third dermatologic opinion for chronic dermatitis affecting her lips, mouth, lower cheeks, and chin since 2017. Previous allergy patch testing due to suspicion for contact dermatitis, revealed multiple allergens including cleure natural lip balm, one of her current products at time of testing. She had self-initiated over the counter 1% hydrocortisone cream for ongoing irritation that continued despite allergen avoidance. Once to twice daily TCS use continued for flares of symptoms over the 5-year period, with escalation of therapy to hydrocortisone 2.5% cream from her previous dermatologist. Flares involved significant erythema plus or minus burning, itching, and crusting. Other treatments such as 6-week supplement discontinuation, continued avoidance of known topical allergens, and elimination of almonds due to food allergen testing yielded no resolution. At the time of evaluation, she was on a regimen of nightly hydrocortisone 2.5% cream and doxycycline 20 mg daily.

No seasonal worsening or atopic history was reported. She endorsed feeling systemically well with no fever, chills, or generalized fatigue.

**Fig 1:**
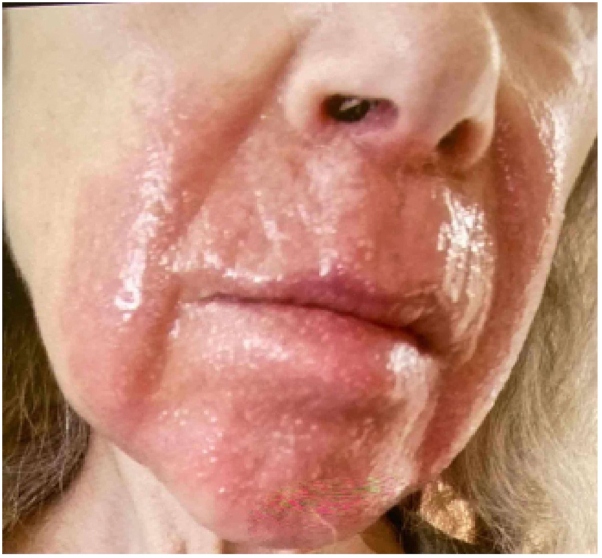
Sharply demarcated bright pink thin plaques overlying the bilateral nasolabial and mid cheeks, upper cutaneous lip, and chin before initial evaluation.

Examination revealed faint pink confluent patches with fine telangiectasias overlying the upper cutaneous lip, edges of the vermilion lip, bilateral nasolabial cheeks, and chin. No secondary scale was seen. Past photos showed sharply demarcated bright pink thin plaques overlying the same currently affected areas (Fig 1). She reported stinging and pain in the affected areas.

Due to TCS history, waxing and waning rash, telangiectasias and sharp demarcation seen in previous photos, a TSW diagnosis was made. Allergic contact dermatitis (ACD) was less suspected due to diligent allergen avoidance. Autoimmune or connective tissue disease was less likely due to lack of systemic symptoms. She was advised to taper nightly TCS use by decreasing frequency of application from 3 to 2 to 1 time per week for a month each and then stopping entirely. Additionally, tacrolimus 0.1% ointment twice daily, continued use of safe product list, and oral doxycycline 20 mg daily for antiinflammatory effect were recommended.

**Fig 2:**
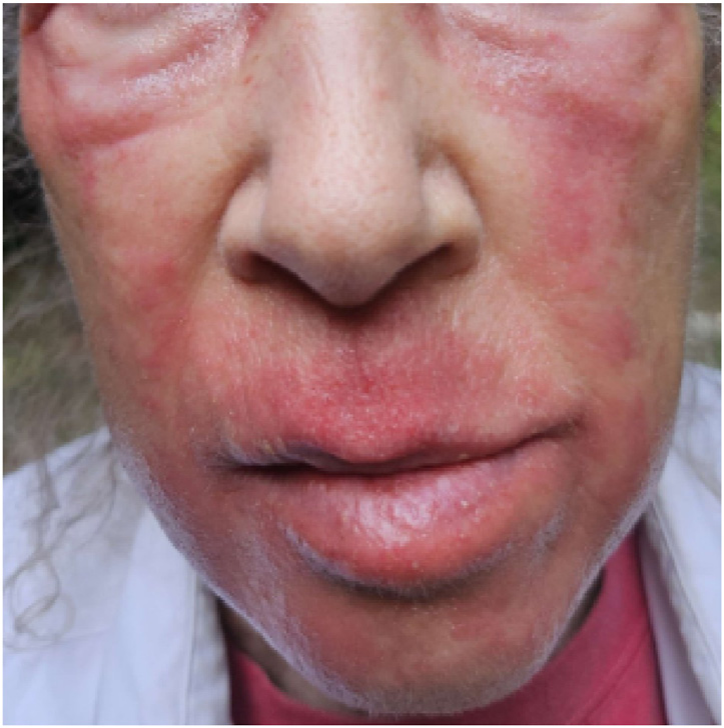
Erythematous flare of the periorbital region, cheeks, lip, and chin following pimecrolimus treatment and initial attempts at TCS taper. *TCS*, Topical corticosteroids.

At subsequent clinic visits, the patient reported improvement but occasional flares requiring 48 hours of TCS usage per flare for control (Fig 2). She also had reactions to both tacrolimus and pimecrolimus topicals during this time reporting erythema, swelling, and burning in areas they were applied, discontinuing use of both. Ruxolitinib 1.5% cream twice daily was prescribed as an alternative antiinflammatory option.

**Fig 3:**
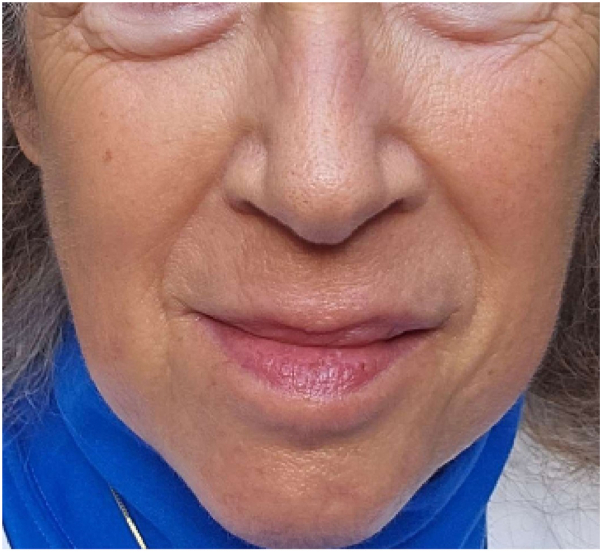
Cleared skin following 3 months of ruxolitinib treatment.

At her 3 month follow-up, she reported once daily ruxolitinib use with substantial improvement with no erythema or plaques noted on provided images or on focused examination (Fig 3). All burning or itching symptoms had resolved, and she had completely discontinued TCS since starting ruxolitinib with no subsequent flares. Continued daily ruxolitinib treatment was recommended due to chronic nature of her condition and likely rebound effect of rash with decreased medication use frequency. At the time of this paper, she has continued topical ruxolitinib 1.5% cream once daily for 5 months with sustained clearance. The plan was to continue to taper off of ruxolitinib cream by changing frequency to 3 times weekly once per day and subsequent further taper over the next several months.

## Discussion

TSW is an increasingly described adverse event characterized by prolonged inappropriate use of TCS leading to erythema and burning upon discontinuation.^1^^,^^2^ Most published cases in existing literature present on the face and genital regions (99.3%), mainly in female (81.0%).^3^ Our patient aligns with the classic presentation, with prolonged TCS use, stinging and pain, affected regions, and gender.

In existing literature, most documented TSW cases follow TCS treatment for atopic dermatitis.^2^^,^^3^ In a retrospective review of 19 TSW patients, 18 had atopic dermatitis, whereas 1 had ACD. Our patient’s initial lip and chin irritation likely stemmed from ACD due to a lip balm identified as a known allergen on her patch testing. This case highlights the possible utility of patch testing for ACD in the setting of TSW.

TSW pathogenesis includes suggested mechanisms such as tachyphylaxis, dysregulation of glucocorticoid receptors, dysregulation of cortisol production by keratinocytes, rebound vasodilation or rebound cytokine cascade secondary to TCS induced barrier impairment.^4^

Treatment protocols lack consensus, but current literature suggests options including discontinuation of TCS with a possible slow taper and various combinations of antihistamines, antibiotics, ice/cool compresses, and psychological support.^3^ This case documents the use of a topical Janus kinase (JAK) inhibitor, ruxolitinib as a treatment for TSW.

JAK inhibitors are increasingly used in atopic dermatitis treatment.^5^ JAK proteins are activated when extracellular ligands bind to type I/II cytokine receptors, recognizing inflammatory mediators such as interleukins and interferons. Once activated, JAK phosphorylates signal transducer and activator of trascription proteins, enabling them to move to the nucleus and initiate downstream gene transcription that leads to continued and increased itching and inflammation.^5^^,^^6^

Considering their role in cytokine pathways, JAK inhibitors may minimize rebound cytokine cascade, a potential TSW mechanism.^4^ Although systemic immune suppressants such as cyclosporine have been used to treat TSW, topical therapy reduces the potential for adverse systemic side effects.

We report this case to expand the clinical toolset employed in treatment of TSW by describing the successful clearing of a chronic, refractory case of TSW with the use of a JAK inhibitor, ruxolitinib.

## Conflicts of interest

Dr Simpson has received personal fees from AbbVie, Amgen, Arcutis, Areteia Therapeutics, Bristol Myers Squibb – BMS, CorEvitas, Corvus, Dermira, Eli Lilly, Evelo Biosciences, FIDE, Forte Bio RX, Galderma, GlaxoSmithKline, Gilead Sciences, Impetus Healthcare, Incyte, Innovaderm Reche, Janssen, Johnson & Johnson, Kyowa Kirin Pharmaceutical Development, Leo, Merck, MJH holding (April 29, 2021), NUMAB Therapeutics AG, Pfizer, Physicians World LLC, PRImE, Recludix Pharma, Regeneron, Roivant, Sanofi-Genzyme, Trevi Therapeutics, and Valeant and has been granted funding or has served as principal investigator for AbbVie, Acrotech, Amgen, Arcutis, ASLAN, Castle, CorEvitas, Dermavant, Dermira, Incyte, Lilly, Kymab, Kyowa Kirin, National Jewish Health, Leo, Pfizer, Regeneron, Sanofi, Target, and VeriSkin. Author Shea and Dr Grinich have no conflicts of interest to declare.
